# OH* 3D Concentration Measurement of Non-Axisymmetric Flame via Near-Ultraviolet Volumetric Emission Tomography

**DOI:** 10.3390/s26010009

**Published:** 2025-12-19

**Authors:** Junhui Ma, Lingxue Wang, Dongqi Chen, Dezhi Zheng, Guoguo Kang, Yi Cai

**Affiliations:** 1 MIIT Key Laboratory of Complex-Field Intelligent Exploration, School of Optics and Photonics, Beijing Institute of Technology, 5 Zhongguancun South Street, Beijing 100081, China; 2 Beijing Institute of Technology, Zhuhai Campus, 6 Jinfeng Road, Tangjiawan, Zhuhai 519088, China

**Keywords:** OH* concentration, flame NUV imaging, VET, non-axisymmetric flame reconstruction

## Abstract

Measuring the three-dimensional (3D) concentration of the ubiquitous intermediate OH* across combustion systems, spanning carbon-based fuels to zero-carbon alternatives such as H_2_ and NH_3_, provides vital insights into flame topology, reaction pathways, and emission formation mechanisms. Optical imaging methods have attracted vital interests due to non-intrusiveness in the combustion process. However, achieving accurate 3D concentration of OH* via imaging in non-axisymmetric flames remains challenging. This work presents a near-ultraviolet (NUV) volumetric emission tomography-based OH* measuring method that integrates a three-layer OH* imaging model, a calibration procedure utilizing narrow-band NUV radiometry, and a threshold-constrained Local Filtered Back-Projection Simultaneous Algebraic Reconstruction Technique (LFBP-SART) algorithm. When applied to a non-axisymmetric Bunsen flame, the method reveals multiple small flame structures matching the fairing pattern in the reconstructed 3D OH* field, with a maximum OH* molar concentration of approximately 0.04 mol/m^3^ and an overall relative uncertainty of about 8.7%. Given its straightforward requirements, this technique is considered adaptable to other free radicals.

## 1. Introduction

With the escalating global warming crisis caused by greenhouse gases, particularly carbon dioxide (CO_2_), zero-carbon fuels such as hydrogen [1] and ammonia [2] are gaining significant attention for their potential in achieving carbon neutrality. However, these fuels face unstable combustion issues like pre-ignition, backfiring, and detonation in hydrogen [3,4], as well as slow flame propagation, high ignition energy, and narrow flammability limits in ammonia [5,6], necessitating further study of their combustion characteristics and mechanisms. Electronically excited hydroxyl radicals, often denoted as OH* or OH-A, are electronic excited forms of ground-state OH(OH-X), exhibiting widely observed self-chemiluminescence in both carbon-based fuels (e.g., hydrocarbons) and zero-carbon fuels (e.g., hydrogen and ammonia). OH* self-chemiluminescence primarily occurs in the near-ultraviolet (NUV) band from 280 nm to 350 nm, peaking at 309 nm, serving as a universal marker for reaction zones and combustion efficiency [7,8]. Density and distribution measurement of OH* in complex combustion or under extreme environments, such as compound combustion, internal combustion engines and aerospace engines, contributes to analysis of flame structure, equivalent ratio, heat release rate, combustion state, and verification of modeling [9,10,11].

Volumetric emission tomography (VET) [12,13] reconstructs three-dimensional (3D) fields by accumulating two-dimensional (2D) line-of-sight (LOS) integral signals of radical chemiluminescence obtained from single or multiple views [14,15,16]. Compared with commonly used combustion-diagnostics techniques that target ground-state OH radicals, such as planar-laser-induced fluorescence (PLIF) [17] and mid-infrared cavity ring-down spectroscopy (MIR-CRDS) [18], VET can provide volumetric information on the 3D OH* distribution within the combustion region. Computed tomography of chemiluminescence (CTC) [12] is a representative implementation of VET, and generally adopts two main 2D image acquisition strategies: divided focal plane [19,20,21] and full focal plane [14,16,22]. In the divided focal plane strategy, one camera simultaneously captures images from multiple views via beamsplitters, optical fibers, or multi-filters, at the expense of spatial resolution and spectral transmittance. In contrast, the full focal plane strategy preserves high spatial resolution and spectral transmittance by capturing multi-view images either simultaneously with multiple cameras or sequentially with a single camera, but at the cost of reduced temporal resolution. Through CTC measurements, a 3D free-radical intensity field can be reconstructed from the 2D integral images, enabling the evaluation of combustion parameters such as flame surface density, wrinkling factor, and flame-normal direction. Researchers including Lv [14], Häber [15], Worth [16], and Anikin [19] have employed CTC to visualize OH* self-chemiluminescence in a variety of flame configurations, thereby demonstrating the capability of CTC to resolve complex flame topologies and serving as a qualitative basis for further quantitative studies. However, most of these studies have remained qualitative or relative because of the inherent LOS integration in the imaging process, whereas quantitative or absolute 3D OH* concentration distributions are crucial for understanding combustion kinetics and for model-based combustion control.

Recently, a few publications [23,24] have attempted to quantitatively measure OH* concentration via VET. Zhao et al. [23] calibrated OH* chemiluminescence from a stable axisymmetric hydrogen–air diffusion flame against the radiance of an integrating-sphere source, establishing a procedure for converting chemiluminescence intensity into absolute OH* concentration. Liu et al. [24] employed customized narrow-band integrating sources at 308 nm and 431 nm to improve calibration accuracy and thereby obtained the simultaneous quantitative densities of OH* and CH* in an axisymmetric methane–air jet diffusion flame, highlighting the importance of spectrally matched calibration for NUV narrowband imaging. These prior quantitative studies demonstrated the feasibility of quantitative VET, and had provided an important technical foundation and motivation for extending VET to more complex, non-axisymmetric flames. However, they have predominantly focused on uniform and axisymmetric combustion flames, and quantitative 3D OH* concentration measurement for non-axisymmetric flames remains a significant challenge.

For axisymmetric combustion targets, when the imaging LOS is perpendicular to the symmetry axis, the resulting 2D projections exhibit a consistent radial intensity distribution at different views. Thus, the 3D field can be reconstructed via Abel inversion, as employed by Zhao [23] and Liu [24]. For non-axisymmetric targets, algebraic tomography algorithms have been widely used for 3D reconstruction of visible radiation from laminar and turbulent flames, including the Algebraic Reconstruction Technique (ART) [25], the Multiplicative Algebraic Reconstruction Technique (MART) [26], and the Simultaneous Algebraic Reconstruction Technique (SART) [27]. The core idea of ART is to iteratively back-project each projection residual along its path and approach the solution via Kaczmarz iterative process. However, under the circumstances of weak signal and high noise level, ART is prone to artifacts such as streaks and rings. SART mitigates streaks and reduces noise sensitivity by view-wise batch updates combined with normalization and relaxation. Based on SART, the Local Filtered Back-Projection–SART (LFBP-SART) [28,29] uses LFBP priors to estimate the target’s effective area and boundary mask, introducing boundary-condition constraints that further improve reconstruction accuracy. However, when the OH* distributed small along certain 2D projection path, the LOS-integrated radiance falls below the camera response threshold, resulting in missing measurements at those views. Traditional LFBP causes missing voxel values along the affected path. To address this issue, we introduce threshold-constraint within the LFBP-SART framework, tailoring the reconstruction to OH* NUV imaging and thereby alleviating data loss and artifacts associated with weak signals.

In this work, we developed a methodology for OH* 3D concentration measurement in non-axisymmetric flames, including design principle, implementation setup and verification. A three-layer model for NUV OH* imaging was established to characterize the emission, transmission, and imaging processes of radicals; radiometric calibration under imaging conditions was implemented in the NUV band to enhance the accuracy of converting grayscale signals to spectral radiance; and a threshold constraint was integrated into the LFBP-SART algorithm to prevent weak NUV signals from being misclassified as reconstruction artifacts. An innovative 360° full-field single-camera rotational imaging setup was constructed to implement and validate the method on a non-asymmetric Bunsen flame, with reconstruction assessment performed through reprojection techniques [30,31] and a proposed cross-validation method in this work. Collectively, this approach demonstrates significant potential for resolving 3D radical concentration distributions in non-axisymmetric combustion systems.

The remainder of this work is organized as follows: the three-layer imaging model and the radical absolute concentration measurement methods are elaborated in Section 2; Section 3 outlines the imaging setup and calibration apparatus; Section 4 presents the measurement of the 3D OH* absolute concentration in the Bunsen flame, and the evaluation results using cross-validation and re-projection methods is given; and Section 5 summarizes the work.

## 2. Theoretical Imaging Model and Quantitative Measurement Method

**Figure 1:**
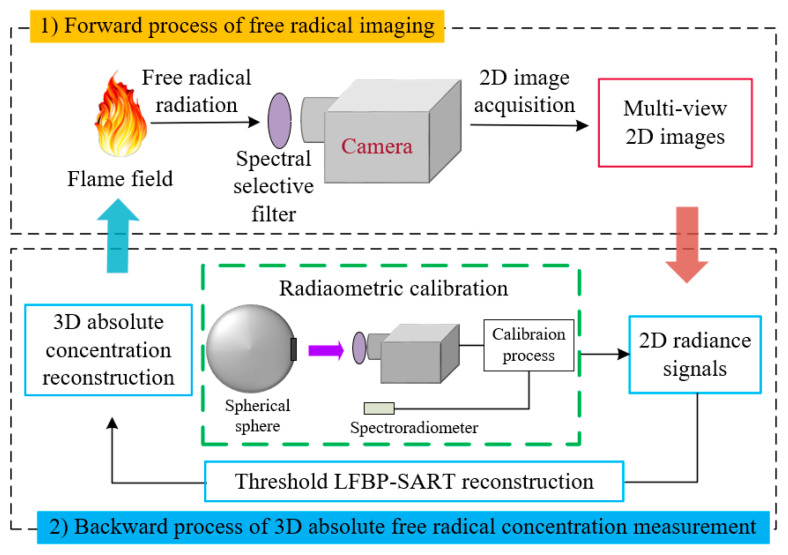
Forward imaging process and backward absolute concentration reconstruction process of free radicals.

This work encompasses two reciprocal processes: the forward process of free radical imaging and the backward process of 3D absolute free radical concentration measurement, as illustrated in Figure 1. In the forward process, we developed a three-layer free radical imaging model that thoroughly delineates the stages of free radical radiation emission, optical system transmission, image sensor response, and the production of the output image. This model forms the theoretical foundation for measuring the absolute concentration of free radicals. The backward process initiates with radiometric correction to convert the grayscale values from the output image into radiance values. Subsequently, the corrected 2D radiance is reconstructed into a 3D radiance distribution using the modified LFBP-SART algorithm. Finally, this 3D radiance distribution is converted into a 3D absolute concentration distribution.

### 2.1. Three-Layer Imaging Model of Free Radicals

**Figure 2:**
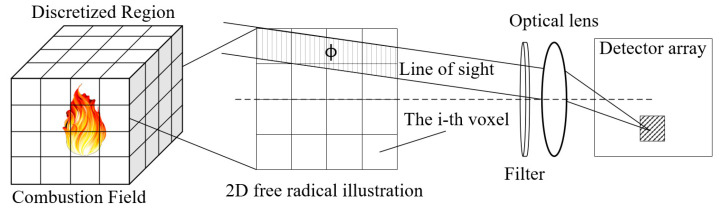
Two-dimensional imaging of 3D free radical field along LOS. Here, *ϕ* denotes the set of voxels intersected by the LOS. The shaded region on the detector array indicates the corresponding response area for the current LOS.

As shown in Figure 2, the radiation field of combustion free radicals is discretized into small voxel units, the discretized resolution can be represented by the edge length d of the voxel unit. During the imaging process, the contribution of different voxels to the detector response can be calculated based on the LOS imaging. A three-layer OH* imaging model was established for the imaging process of single voxel free radical. During the propagation of flame radiation, typical species that cause absorption and scattering include soot particles and major combustion products such as CO_2_ and H_2_O. In the present study, the target flame is non-sooting, and soot particles can therefore be neglected. A narrow-band filter confines the imaging to the 305–315 nm spectral range. Within this band, the absorption of NUV radiation by the major combustion products is negligible, both gas-phase absorption and scattering can be ignored in this model. Therefore, the model is underpinned by the following two simplifying assumptions that streamline the imaging process of free radicals: the radiation losses that might occur due to particle scattering and absorption within the combustion field were neglected; the imaging distance was assumed to far exceed the system’s focal length, and the parallel rays were taken to approximate the imaging process.

**Figure 3:**
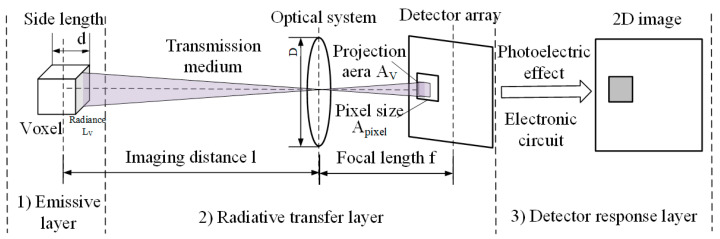
Single voxel three-layer imaging model.

Given the above assumptions, the collective imaging of multiple free radical voxels can be decomposed into the superimposed images of their individual imaging counterparts. As illustrated in Figure 3, the imaging process for a single voxel element can be conceptualized using a three-layer imaging model. Build on the standard “source–path–sensor” image formation process, this model is specialized and parameterized for NUV OH* tomography by explicitly incorporating the radical radiation, spectral transmittance of the optical path, the response of CMOS sensor, and the integrating-sphere-based calibration procedure. This specialization enables a quantitative voxel-to-gray value mapping required for reconstructing absolute 3D OH* concentration in non-axisymmetric flame. Firstly, the radiation of free radical is initiated in the emissive layer. The radiation of the first layer then transmits into the transfer layer. In this layer, radiations traverse from the combustion field to the detector surface through transmission medium (air in this case), and an optical system composed of filters and lenses. Finally, in the detector response layer, the incident photons are converted into grayscale signals by the detector.

In the free radical emissive layer, the radiation intensity of a single voxel, with a side length of *d*, is directly proportional to the concentration of free radicals within the voxel. For free radicals denoted as N*, the symbol C_N*_ represents the molar concentration of free radicals per unit volume. The radiance *L_V_* of a single voxel per unit time and unit solid angle can be expressed as follows:

(1) $$L_{V}=\int_{\lambda_{1}}^{\lambda_{2}}\frac{hcAC_{N^{*}}N_{A}V}{\lambda 4\pi S}d\lambda$$

where *L_V_* is the radiance emitted by one voxel, *h* the Planck constant, *c* the speed of light, *λ* the radiation wavelength, *A* the Einstein coefficient, *N_A_* the Avogadro constant, *V* the volume of single voxel, *S* the voxel surface area, and *λ*_1_ to *λ*_2_ represents the spectral range.

The radiative transfer layer serves as the conduit for transporting the three-dimensional radiation emitted by free radicals from its emissive surface to the camera detector’s surface. Within this layer, the three-dimensional radiation emitted by the free radicals is captured and converted into a 2D image. The intensity *P_V_* of radiation received by a single pixel from a voxel per unit time can be expressed as follows:

(2) $$P_{V}=\tau L_{V}\Omega A_{V}=\Omega A_{V}\int_{\lambda_{1}}^{\lambda_{2}}\tau_{m\lambda }\tau_{p\lambda }\tau_{o\lambda }\tau_{\lambda }\frac{hcAC_{N^{*}}N_{A}V}{\lambda 4\pi S}d\lambda$$

(3) $$\tau =\int_{\lambda_{1}}^{\lambda_{2}}\tau_{m\lambda }\tau_{p\lambda }\tau_{o\lambda }\tau_{\lambda }d\lambda$$

where *τ* is the total transmittance of the radiative transfer layer, including the spectrum transmittance of the transmission medium *τ_mλ_*, the optical path *τ_pλ_*, the optical lens *τ_oλ_*, and narrow-band NUV wavelength selective filter *τ_λ_*. *A_V_* is the receptive area on a single pixel of a single voxel. The total solid angle *Ω* represents the proportion of the radiation collected by the detector through the optical aperture *D* and can be approximated as follows under parallel ray assumption:

(4) $$\Omega =\frac{1}{4}\pi {\left(\frac{D}{f}\right)}^{2}$$

where *f* represents the optical focal length of the imaging lens.

The radiation received by the detector surface is converted into a grayscale image output in the response layer. In this layer, radiation undergoes photoelectric conversion and electron multiplication (in enhanced detectors) before being read out as a grayscale signal by the electronic circuit. This process can be described as follows:

(5) $$G=G_{0}+K\eta \int \int_{\lambda_{1}}^{\lambda_{2}}\frac{P_{V}\lambda }{hc}d\lambda dt$$

where *G*_0_ is the response offset under dark background, *K* the conversion coefficient of electrons to gray value, and *η* the total quantum efficiency of the detector in the UV band, which is the integral value of the quantum efficiency *η_λ_* as follows:

(6) $$\eta =\int_{\lambda_{1}}^{\lambda_{2}}\eta_{\lambda }d\lambda$$

Assuming that the concentration of free radicals of a single voxel remains constant within the exposure time *t_e_*, the Equation (6) can be simplified as follows:

(7) $$G=G_{0}+K\eta t_{e}\int_{\lambda_{1}}^{\lambda_{2}}\frac{P_{V}\lambda }{hc}d\lambda$$

In the imaging process, the radiation detected by one pixel does not originate from a single voxel but is an aggregate of contributions from multiple voxels along the LOS instead. This cumulative effect can be mathematically represented as an integral or summation over all the voxels intersecting with the LOS, reflecting the collective influence of these voxels on the detected intensity of pixel *j*. The expression for *G_pj_*, is as follows:

(8) $$G_{pj}=G_{0}+\tau \Omega K\eta t_{e}A_{pixel}\int_{\lambda_{1}}^{\lambda_{2}}\frac{\lambda }{hc}d\lambda \cdot \sum_{V_{i}\subset \phi }w_{ij}L_{V_{ij}},1\leq i\leq N,1\leq j\leq p\times (m\times n)$$

where *G_p_* is the grayscale output of the target pixel, *w_i_* is the projection coefficient of *i*-th voxel to *j*-th pixel, *N* the total number of voxels, *p* the total number of views, and *m* × *n* the resolution of 2D image. The summation term in the above equation represents the total voxel radiance onto a single pixel. To facilitate calculation, this term is extracted and referred to as the collective radiance *L_sum_*:

(9) $$L_{sum}=\sum_{V_{i}\subset \phi }w_{ij}L_{V_{ij}}$$

### 2.2. Absolute Concentration Measurement Based on Radiometric Calibration and Threshold-Constrained LFBP-SART

In this section, we use radiometric calibration and tomographic reconstruction to establish a quantitative relationship between the 2D NUV images and the 3D spatial voxel radiance of the combustion field, from which the voxel-resolved concentration is subsequently obtained. The 3D reconstruction is based on the existing LFBP-SART, augmented with a threshold constraint tailored to the low-signal characteristics of the NUV band, which effectively suppresses reconstruction artifacts while avoiding the loss of low-intensity features.

Radiometric calibration is necessary to determine the conversion coefficient between the grayscale signal and the radiance of free radicals. To ensure accuracy, calibration is typically conducted under the same imaging conditions as the 2D image acquisition process. These conditions include identical focal length, imaging distance, system transmittance, and spectral range. During calibration, the imaging system is calibrated using a standard light source with known radiance *L_s_* in the imaging bands *λ*_1_–*λ*_2_. The detector’s response to the standard light source at a single pixel *G_s_* can be expressed as follows:

(10) $$G_{s}=G_{0}+\tau \Omega K\eta t_{e}L_{s}\int_{\lambda_{1}}^{\lambda_{2}}\frac{\lambda }{hc}A_{pixel}d\lambda =G_{0}+K_{system}L_{s}$$

(11) $$K_{system}=\tau \Omega K\eta t_{e}\int_{\lambda_{1}}^{\lambda_{2}}\frac{\lambda }{hc}A_{pixel}d\lambda$$

*K_system_* is considered as a constant value under the same imaging conditions. Thus, the flame radiation on single pixel can be abbreviated as:

(12) $$G_{p}=G_{0}+K_{system}L_{sum}$$

Therefore, the collective radiance *L_sum_* along the line of sight corresponding to the target pixel can be deduced as follows:

(13) $$L_{sum}=\frac{G_{p}-G_{0}}{K_{system}}$$

After radiometric correction, the collective radiance is further transformed into a 3D spatial concentration distribution using a 3D reconstruction algorithm. ART is renowned for its excellent field reconstruction capabilities and is widely used in flame field reconstruction. In the NUV band, however, ART exhibits poor robustness to significant noise interference, which is introduced by selective narrowband filters. SART incorporates the advantages of both convergence speed and reconstruction accuracy. Unlike ART, which accumulates ray errors one direction at a time, SART accumulates all ray errors in one direction simultaneously, achieving a better noise smoothing effect and thus offering improved resistance to noise interference. The algorithm is as follows:

(14) $$L_{V_{i}}^{\left(t+1\right)}=L_{V_{i}}^{\left(t\right)}+\xi \frac{\sum_{j\in P_{\Phi }}w_{ij}\Delta_{ij}}{\sum_{j\in P_{\Phi }}w_{ij}},1\leq i\leq N,1\leq j\leq p\times (m\times n)$$

(15) $$\sum_{j\in P_{\Phi }}w_{ij}\Delta_{ij}=\sum_{j\in P_{\Phi }}\left[w_{ij}\frac{L_{sum_{j}}-\sum_{i}w_{ij}L_{V_{i}}^{\left(t\right)}}{\sum_{i}w_{ij}}\right]$$

where *t* represents iterations, *N* the total number of voxels, *p* the total number of views, *m* × *n* the resolution of projection image, *ξ* relaxation factor, *P_ϕ_* the set of rays that intersect with *i*-th voxel in a given view, and ${∆}_{ij}$ symbolizes the orthographic projection and correction process.

In addition to noise interference, the artifacts introduced by reconstruction algorithms cannot be avoided. Xu [28] utilized LFBP algorithm to address this issue in fluid monitoring applications. Hossain [29] adapted LFBP for flame reconstruction, combining it with the SART to delineate the contour of the reconstruction area. This method effectively mitigated the artifact problem typically caused by reconstruction algorithms. LFBP is developed by employing logical operations based on FBP, implementing the inverse Radon transform according to the projection-slice theorem. The FBP process can be expressed as follows:

(16) $$\left[\begin{array}{l} f(\bar{x})\approx \frac{\pi }{p}\sum_{k=1}^{p}Q_{\theta_{k}}(\bar{x}\cdot \bar{n})\\ Q_{\theta_{k}}(\bar{x}\cdot \bar{n})=(h*P_{\theta })(x\prime ) \end{array}\right.$$

where $Q_{\theta_{k}}$ represents the filtered projection, and the filtering process is completed through the filtering function *h*. $\bar{x}$ the projection beam vector, and $\bar{n}$ the unit vector normal to the projection beam. LFBP calculation replaces the summation process with a logical operation:

(17) $$f(\bar{x})\approx \frac{\pi }{p}\overset{p}{\underset{k=1}{\Theta }}Q_{\theta_{k}}^{\prime }(\bar{x}\cdot \bar{n})$$

The operational logic for *Θ* is

(18) $$C=A\Theta B\;c_{i}=\left\{\begin{array}{l} 0,\;if\;a_{i}=0\cup b_{i}=0\\ 1,\;if\;a_{i}=b_{i} \end{array}\right.$$

and discrimination for $Q_{\theta_{k}}^{\prime }$ is

(19) $$Q_{\theta_{k}}^{\prime }=\left\{\begin{array}{l} 1,\;if\;Q_{\theta_{k}}(\bar{x}\cdot \bar{n})\geq 0\\ 0,\;if\;Q_{\theta_{k}}(\bar{x}\cdot \bar{n})<0 \end{array}\right.$$

The premise of the above equation assumes that the radiation intensity of each voxel is sufficiently high. This assumption holds when there are no other voxels along the ray path, allowing the radiation integral value of an individual voxel to exceed the detection limit of the detector and trigger a response. In the visible bands, this assumption is reasonable. However, in the NUV band, achieving sufficient integration of OH* luminescence intensity from individual voxels to satisfy this assumption is challenging, often resulting in the loss of weak OH* signals. To address this issue, a threshold constraint was introduced to the discrimination process as follows:

(20) $$c=\left\{\begin{array}{l} 0,\;if\;\mathrm{sum}(Q^{\prime })/p<\mathrm{th}\\ 1,\;if\;\mathrm{sum}(Q^{\prime })/p\geq \mathrm{th} \end{array}\right.$$

where *th* (0 ≤ th ≤ 1) represents threshold, sum($Q^{\prime }$) the sum of filtered back-projection logic values in each direction for the target voxel, sum($Q^{\prime }/p$) the proportion of back-projection logic values being 1 in each projection direction. When a smaller threshold is used, low-intensity voxel details are preserved more completely, but noise-induced artifacts are also retained. As the threshold increases, the suppression of noise artifacts becomes stronger. However, when the threshold is too large, low-intensity voxel details are unavoidably lost. Based on numerical simulations of reconstructions performed for different noise levels and threshold values (see the Appendix A.2 for details), a threshold in the range 0.45–0.55 was found to provide a more reasonable balance between artifact suppression and detail preservation. During the reconstruction process, the improved LFBP algorithm was used to overlay contour information on the results obtained from the SART reconstruction, which can be expressed as follows:

(21) $$w_{ij}L_{V_{i}}=0,\;if\;c_{ij}=0$$

Furthermore, the voxel radiance *L_v_* can be obtained through the reconstruction algorithm. Then the concentration of a single voxel can be expressed as follows:

(22) $$C_{N^{*}i}=\frac{4\pi L_{V_{i}}S}{N_{A}\int_{\lambda_{1}}^{\lambda_{2}}hc/\lambda AVd\lambda },1\leq i\leq N$$

## 3. Experimental Setup

### 3.1. Two-Dimensional Imaging Setup for Bunsen Burner Flame

**Figure 4:**
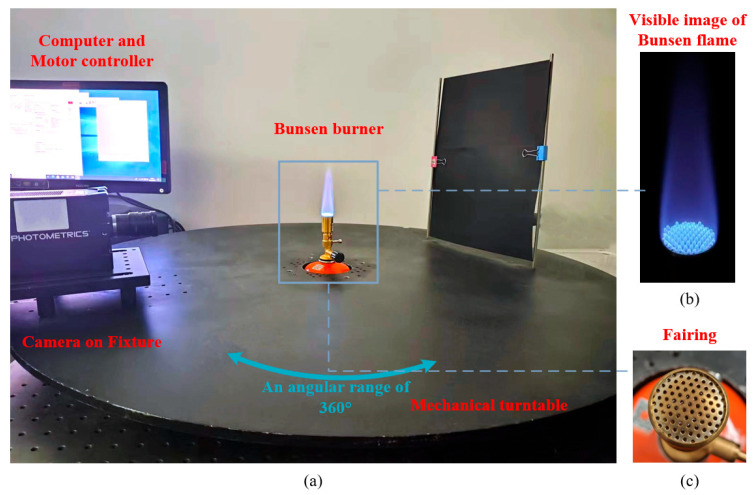
Full-angle single camera rotation time-resolved OH* self-chemiluminescence imaging system: (**a**) the imaging setup, consisting of a mechanical turntable, a motor controller, a fixture, a camera, and a computer; a Bunsen burner is positioned at the center; (**b**) the visible image of Bunsen flame, with small flame structure arranged corresponding to the pattern of fairing, and (**c**) the periodic pattern of circular holes of the fairing.

**Figure 5:**
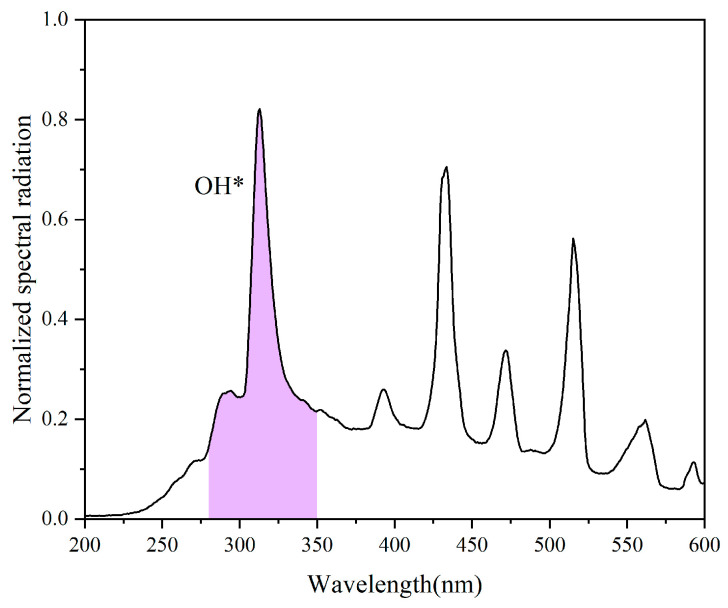
Normalized spectral radiation of Bunsen flame at a wave range of 200–600 nm, measured by AvaSpec-HSC-TEC. The purple region highlights the primary wavelength band of the OH* emission peak.

The setup employs a single-camera rotation imaging scheme, consisting of an NUV camera mounted on a rotational turntable, as depicted in Figure 4, to capture 2D images of a Bunsen burner flame. The fuel is a mixture of isobutane and propane. The nozzle of the Bunsen burner is covered by a fairing with a periodic pattern of circular holes, with a diameter of 1 mm, and center distance of 2 mm, as shown in Figure 4c. These circular holes create multiple small flame distributions, referred to for convenience as small flames, resulting in a non-axisymmetric flame structure. The normalized spectral radiation of the Bunsen flame within the wavelength range of 200 nm to 600 nm was measured using an Avantes spectrometer (AvaSpec-HSC-TEC, Avantes, Apeldoorn, The Netherlands). As depicted in Figure 5, the spectrum exhibits strong emission peaks between 305 nm and 315 nm, indicating the presence of OH* of Bunsen burner flame.

To selectively capture the radiation from OH*, a narrowband NUV filter with a center wavelength of 310 nm (FWHM 10 nm) was installed in front of the lens. At this wavelength, CO_2_* and soot radiation are the primary interference factors. However, according to Wang [32], the radiation of CO_2_* in the NUV band constitutes approximately 3.5% of the OH* radiation intensity, which is negligible. Furthermore, Yang [33] notes that alkane flames do not exhibit strong soot emissions in the NUV region, rendering them negligible compared with OH* radiation. Therefore, the energy detected in this band can be attributed entirely to OH* self-luminescent radiation, and its intensity directly reflects the quantity and distribution of OH*.

A high-speed motor-driven mechanical turntable was utilized for full-angle rotation imaging of the flame. This turntable features a 360° rotation range. It has a diameter of 1 meter, with a 100 mm circular hole at the center designed to mount the combustion device. During rotation, the Bunsen burner remains stationary at the center, ensuring consistent imaging conditions. For capturing 2D flame images, the Prime 95B CMOS camera from Photometrics (Tucson, AZ, USA), with BSI enhancement in the NUV band, was selected. The camera’s specifications are detailed in Table 1. An NUV lens (AZURE Technology, Cambridge, UK), with a focal length of 50 mm, an entrance pupil diameter of 34 mm, and a spectral transmittance range of 200–1000 nm, was mounted on the camera. A camera fixture with an adjustable imaging distance was installed to maintain a consistent 30 cm distance from the flame. This setup obviated the need for complex optical splitter systems, thereby enhancing resolution and reducing energy attenuation in the flame projection. To mitigate blur caused by a shallow depth of field, the aperture was adjusted down to a stop diameter of 2 mm to limit the radiation entrance. For the current system, this aperture provides a 28 mm depth of field under the NUV band, sufficient to cover the target flame region (20 mm). To capture the flame’s projection from multiple views, 36 images were taken over a 180° span, each with an integration time of 20 ms and an angular increment of 5°. During acquisition, the camera was rotated by a motor controller in 5° steps from 0° to 175°, dwelling 2 s at each position for the exposure.

### 3.2. Radiometric Calibration System

**Figure 6:**
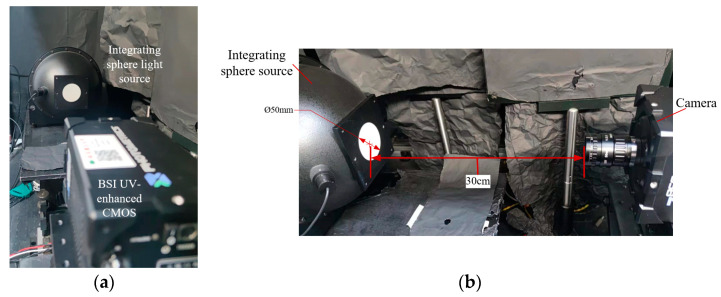
The front view (**a**) and side view (**b**) of the radiance calibration setup.

The radiometric calibration system, depicted in Figure 6, utilizes an integrating sphere light source with an outlet diameter of 50 mm, serving as a uniform planar light source for radiometric calibration. This calibration process involves calculating the camera’s response coefficient to radiance and performing flat-field correction. During the calibration, a filter was installed in front of the lens, with its transmittance integrated into *K_system_* in Equation (11). This selective filter ensures that the detector responds exclusively in the NUV band, thereby eliminating interference from other bands. To reduce noise, an average of 100 images were taken. The *K_system_* value was determined by calculating the average gray value of the central 10 × 10 pixel region, which serves as the standard response to the uniform planar light source. In this work, following calibration and calculation, the system coefficient *K_system_* was determined to be 527.97 (1/(μW·cm^−2^·sr^−1^)) (with uncertainty of 3.2%, see the Appendix B for details). The flat field correction coefficient *F_j_* for each pixel is calculated by the ratio of the single pixel’s grayscale response *G_pj_* to the standard response *G_avr_*:

(23) $$F_{j}=\frac{G_{pj}-G_{0}}{G_{avr}-G_{0}}$$

where *G*_0_ is the offset gray value in the absence of radiation.

## 4. Results and Discussion

### 4.1. Reconstruction Results

**Figure 7:**
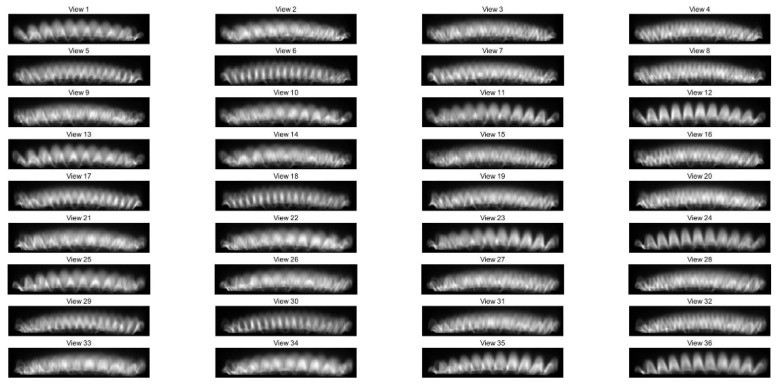
Original NUV 2D flame images from 36 views.

**Figure 8:**
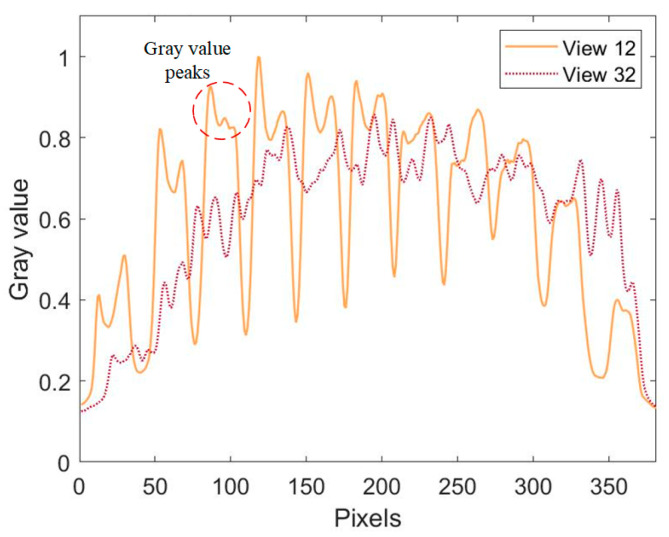
Normalized gray values distribution at HAB 2.208 mm from different views.

To further validate the proposed concentration reconstruction method, we used the setups described in Section 3 to perform 3D OH* reconstruction and quantization of Bunsen burner flames. Using 3D-phantom-composed annularly distributed Gaussian functions, we evaluated the performance of the reconstruction algorithm under different view numbers. The results indicate that the imaging accuracy improves with increasing view number (see the Appendix A.1 for details). In this study, 36 projection views are employed for the reconstruction in order to achieve high quantitative accuracy. Figure 7 displays 2D images captured from 36 different views. Predominant OH* emission was observed near the nozzle of the Bunsen burner, with weaker emissions noted at higher distances above the nozzle. To analyze the characteristics of these 2D images, data from the 46th row (height above burner, HAB, 2.208 mm) of image data from the 12th and 32nd views were used to draw the grayscale value curve depicted in Figure 8. The multiple peaks on these curves suggest that the flame consists of several independent small flames, created by the fairing. Different views reveal varying patterns of OH* emission projected onto the detector along different LOS. For instance, on the 12th view, 11 distinct peaks are clearly distributed along the line. Conversely, on the 32nd view, the grayscale peaks are less distinguishable, indicative of intersections and overlaps of the small flames.

Before the reconstruction, both radiance calibration and flat field correction were conducted using the radiometric calibration system. Subsequently, Zhang’s method was employed to correct camera imaging errors. The angular rotation and offset errors introduced by the rotation of the turntable were corrected by aligning the position of the Bunsen burner itself. Since the fairing is curved, the section between its vertex and bottom partially obstructs the field of view, resulting in images on certain views cannot be captured. According to the work of Liu [34], the data loss will cause error in reconstruction. To avoid the loss of data, the reconstruction target area lay above the vertex of the Bunsen burner, which is the region between HAB 1.173 mm to HAB 4.347 mm. The target volume comprised 270 × 270 × 46 voxels, corresponding to a target area of 26.46 mm × 26.46 mm × 3.174 mm, with the voxel horizontal resolution of 0.098 mm/voxel and vertical resolution of 0.069 mm/voxel. All reconstructions were performed on a workstation equipped with an Intel(R) Core(TM) i9–9900K CPU, an NVIDIA GeForce RTX 2070 SUPER GPU, and MATLAB R2019b; under the present configuration, a single 3D reconstruction required approximately 14 h of computation time. The origin of the reconstruction was positioned at the bottom center of the reconstructed volume. The maximum number of iterations was set to 600, and the iteration termination condition was defined as follows:

(24) $$\sigma \leq 10^{-6},\;where\;\sigma =\frac{\sum_{i=1}^{N}\Delta f_{i}}{\sum_{i=1}^{N}f_{i}^{\left(t\right)}}=\frac{\sum_{i=1}^{N}|f_{i}^{\left(t+1\right)}-f_{i}^{\left(t\right)}|}{\sum_{i=1}^{N}f_{i}^{\left(t\right)}},1\leq i\leq N$$

**Figure 9:**
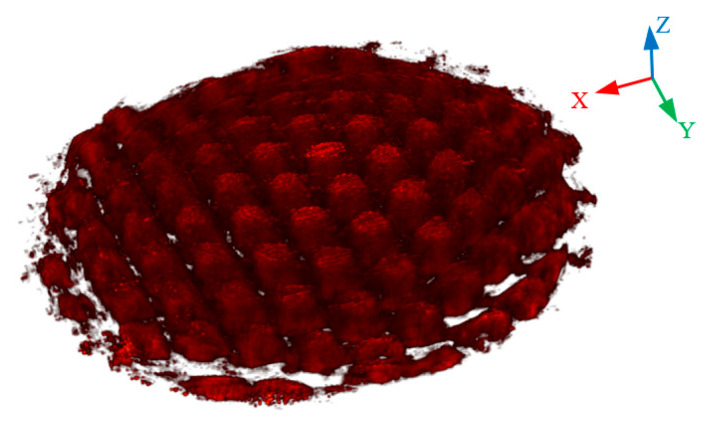
OH* 3D distribution contour of the reconstruction results.

**Figure 10:**
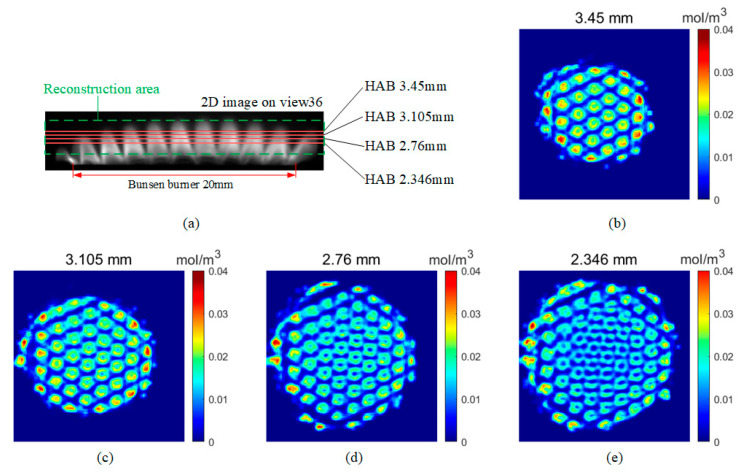
Horizontal slices of OH* distribution on different HAB: (**a**) schematic diagram of the reconstructed area and the location of the horizontal slice; (**b**–**e**) horizontal slices on HAB 3.45 mm, 3.105 mm, 2.76 mm and 2.346 mm, respectively.

**Figure 11:**
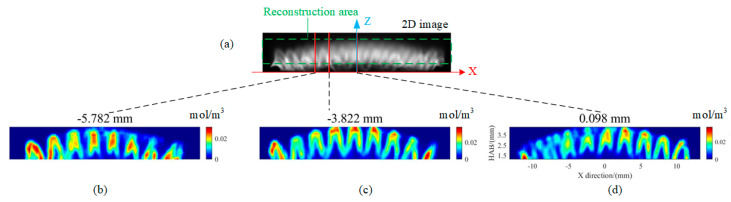
Vertical slices of OH* distribution on different location: (**a**) schematic diagram of the reconstructed area and the location of the vertical slice; (**b**–**e**) vertical slices on different plane Y of −5.782 mm, −3.822 mm, and 0.098 mm, respectively.

The OH* distribution contour obtained from the reconstruction results is shown in Figure 9. The OH* distribution revealed multiple small arch-shaped structures, with OH* concentrated at the edges and near-zero concentration at the inner space under the arch-shaped structures. This distribution of small flames corresponds well with the structure of the Bunsen burner fairing. The horizontal slices and vertical slices of the reconstruction results are shown in Figure 10 and Figure 11, respectively.

In the horizontal sections as shown in Figure 10, the OH* emission displayed an annular pattern that aligned with the fairing’s pattern. Due to the curved shape of the fairing, the flame ring at the same height appeared varying diameters and density values. In the vertical slices as depicted in Figure 11, the number of small flames fluctuates based on their position relative to the distribution of fairing circles. For instance, there were fewer small flames near the edge of the burner and more near the center. From the reconstructed slices, the OH* is predominantly localized near the flame edges, forming arch-shaped structures with a maximum concentration value of approximately 0.04 mol/m^3^, while the slice-averaged OH* concentrations are 0.0040, 0.0054, 0.0066, and 0.0077 mol/m^3^ for the four slices, respectively. Below the arches, an extremely low OH* distribution, nearing zero, was observed. This arch-shaped distribution arises because the fuel jet issuing from the Bunsen-burner fairing mixes most intensely with the surrounding air in the shear layer near the jet boundary. As a result, the mixture near the edge reaches flammable (near-stoichiometric) conditions first, leading to stronger reaction rates and thus enhanced OH* concentration along the arch. In contrast, the region below the arch is closer to the faring, where the fuel is still relatively unmixed and fuel-rich, so combustion is weaker or has not yet occurred and the OH* level remains low.

### 4.2. The Evaluation of Flame Reconstruction

Since the free radical concentration data cannot be obtained by direct measurement, the reconstruction accuracy was evaluated by both cross-validation of different reconstruction results and re-projection methods. The cross-validation method takes 2D images of different views to implement reconstructions, and then the correlation coefficient of reconstruction results from two sets of data were calculated. The specific calculation of the correlation coefficient is:

(25) $$R_{\alpha -\beta }=\frac{\sum_{i=1}^{N}\left(f_{i}^{\alpha }-\bar{f^{\alpha }})\times (f_{i}^{\beta }-\bar{f^{\beta }}\right)}{\sqrt{\sum_{i=1}^{N}{\left(f_{i}^{\alpha }-\bar{f^{\alpha }}\right)}^{2}}\times \sqrt{\sum_{i=1}^{N}{\left(f_{i}^{\beta }-\bar{f^{\beta }}\right)}^{2}}}$$

where *α* and *β* represent two different sets of 3D reconstruction work, which the 2D projections used for reconstruction come from different views of data, *f_i_^α^* and *f_i_^β^* represent the reconstructed 3D distribution of two sets of work, and $\bar{f^{\;\alpha }}$ and $\bar{f^{\;\beta }}$ are the average values of the two reconstructed 3D fields. The re-projection method uses the reconstructed 3D field to re-project the 2D projection, which did not participate in the reconstruction process. The error between re-projection and the 2D images obtained by experimental acquisition can be expressed as follows:

(26) $$E_{re-proj}=\frac{||p^{re-proj}-p^{true}|{|}_{2}}{||p^{true}|{|}_{2}}$$

where *p^re-proj^* and *p^true^* are the re-projection and the true 2D image acquired by the imaging setup, respectively.

**Figure 12:**
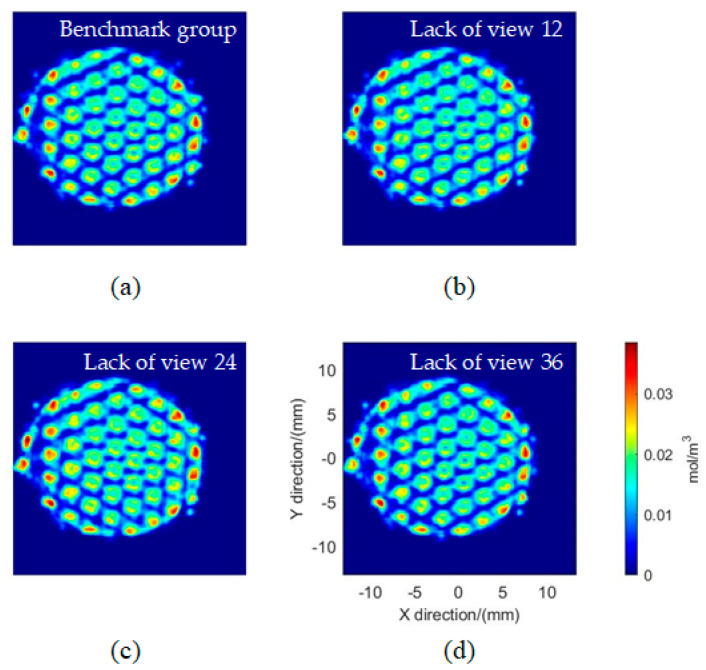
Comparison of horizonal OH* distribution slices on HAB 3.105 mm for different reconstructions: (**a**) benchmark group; (**b**–**d**) different groups with 35 views lack of view 12, view 24 and view 36, respectively.

Three groups of reconstruction work were implemented. Each group of data consists of 2D images from 35 views. View angles 12, 24, and 36 are selected as verification images for re-projection error calculation in the three groups, without participating in the reconstruction process, respectively. The result reconstructed by 36 views in Section 4.1 was used as the benchmark group, and its correlation coefficient with the other three groups of reconstruction results is calculated, respectively. The correlation coefficients of the three groups are 0.9211, 0.8963, and 0.9408, respectively, with an average value of 0.9194. Combining this reconstruction accuracy with the 3.2% (see the Appendix B for details) uncertainty of the radiometric calibration and using a root-sum-square estimate, the overall relative uncertainty of the reconstructed OH* concentration is approximately 8.7%. The slice comparison of the results and the benchmark group is shown in Figure 12. Under the reconstruction work of different view data, the 3D distribution results maintain good similarity.

**Figure 13:**
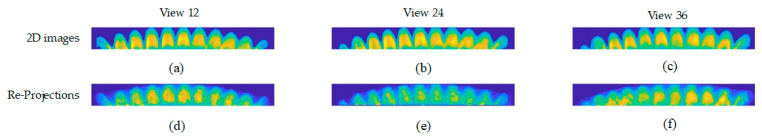
Comparison of captured 2D images and the corresponding reprojections: (**a**–**c**) captured 2D images of the setup from view 12, 24 and 36; (**d**–**f**) reprojections of view 12, 24 and 36, each reprojected from different group reconstructed with 35 views lack of view 12, view 24 and view 36, respectively.

The 3D results obtained by the three sets of reconstruction works are further used to re-project views 12, 24 and 36, respectively. The results are shown in Figure 13. The errors of the three sets between re-projections and captured images are 0.2746, 0.3751 and 0.2902, respectively. Both correlation coefficients of cross-validation and re-projection errors prove the reconstruction algorithm has good credibility.

## 5. Conclusions

In summary, this work proposed and demonstrated, for the first time to the best of our knowledge, an NUV VET method for quantitative reconstruction of the 3D OH* concentration field in a non-axisymmetric flame. Two-dimensional NUV images of a non-axisymmetric Bunsen burner flame from 36 views over 180°, captured by a single-camera rotational imaging setup, were used to carry out the reconstruction through threshold-constrained LFBP-SART algorithm. This method successfully reconstructed the periodically distributed small flames of the Bunsen flame, revealing that OH* was distributed in an arched shape, with maximum concentrations of about 0.04 mol/m^3^ at the arch’s edge and near zero at the center bottom. This method provides a practical, cost-effective solution for measuring 3D non-axisymmetric radical concentration with low environmental requirements. However, due to the setup’s time-resolved imaging characteristics, image acquisition at different views were performed sequentially, which means the system is better suited to measure stable flames or the period-averaged distribution of periodic flames. The present study focuses on statistically steady flames, for which multiple views can be acquired sequentially using a single NUV camera with rotation. The three-layer imaging model and the threshold-constraint LFBP–SART reconstruction framework are in principle applicable to unsteady or transient flames as well; however, in that case, simultaneous multi-view acquisition would be required. This could be achieved by employing multiple synchronized NUV cameras or optical beam-splitting components to capture all views simultaneously, at the expense of increased experimental complexity, more demanding cross-camera radiometric calibration, and potential reductions in signal-to-noise ratio due to beam splitting. Additionally, when extending the present framework to other fuel combustion scenarios, such as hydrogen and ammonia flames, the inherent OH* concentration levels of the target flame must be taken into account and the overall measurement strategy should be adjusted accordingly. For instance, in NH_3_–H_2_ mixed combustion, the relatively low reactivity of NH_3_ leads to reduced OH* formation and thus weaker spontaneous NUV emission, so high-gain NUV imaging through an image intensifier would be required to maintain a sufficient signal-to-noise ratio, while potential spectral interference from other radicals such as NH* would also need to be considered. The proposed framework can also be extended to other chemiluminescent radicals such as NO*, NH* and CH* within a multi-species tomographic method, although this will require improved detection sensitivity and multi-spectrum acquisition schemes. These extensions constitute an important direction for our future work.

## Figures and Tables

**Table 1:** Camera parameters of Prime 95B CMOS.

Sensitivity Range	Pixel Size	Highest Resolution	Quantum Efficiency
200–1000 nm	11 μm × 11 μm	1200 × 1200	35%@310 nm

**Figure A1:**
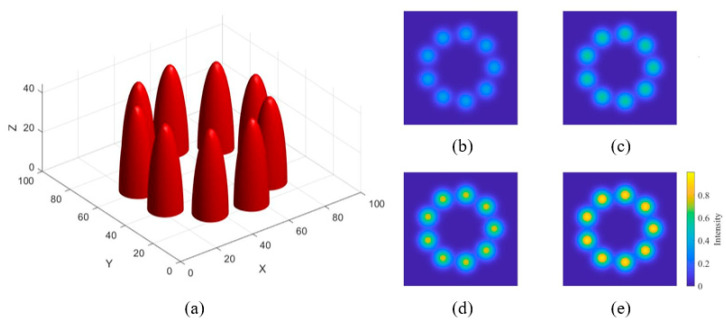
(**a**) 3D annularly distributed Gaussian functions; (**b**–**e**): 2D intensity slices at layers 10, 20, 30, and 40, respectively.

**Figure A2:**
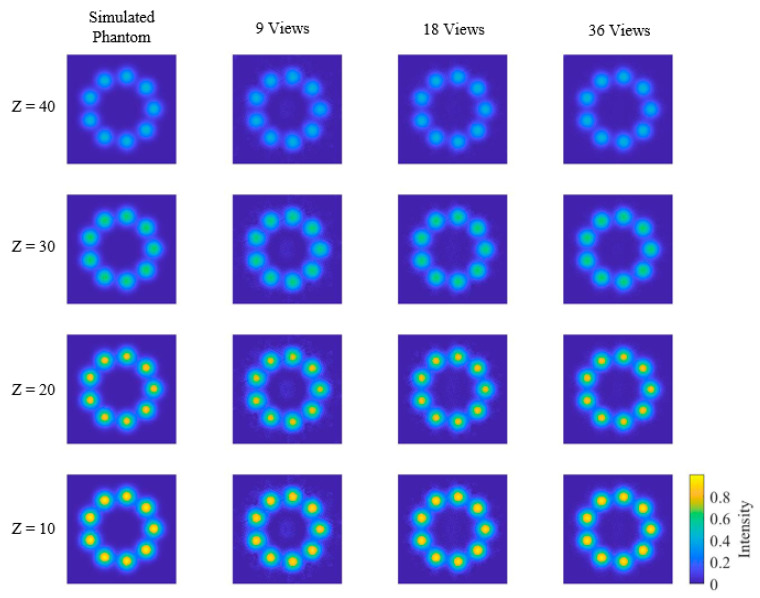
Horizontal slices comparison of simulated phantom and reconstruction results of different view numbers. From left to right: simulation data and reconstruction results using 9, 18, and 36 perspectives. From top to bottom: horizontal slices at Z = 40, 30, 20, and 10.

**Figure A3:**
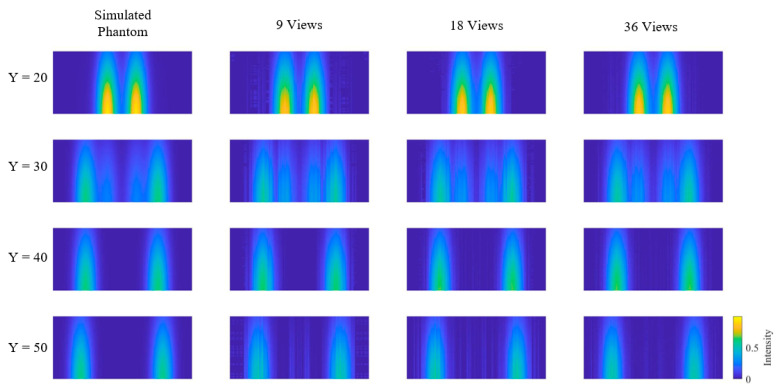
Vertical slices comparison of simulated phantom and reconstruction results of different view numbers. From left to right: simulation data and reconstruction results using 9, 18, and 36 perspectives. From top to bottom: horizontal slices at Y = 20, 30, 40, and 50.

**Figure A4:**
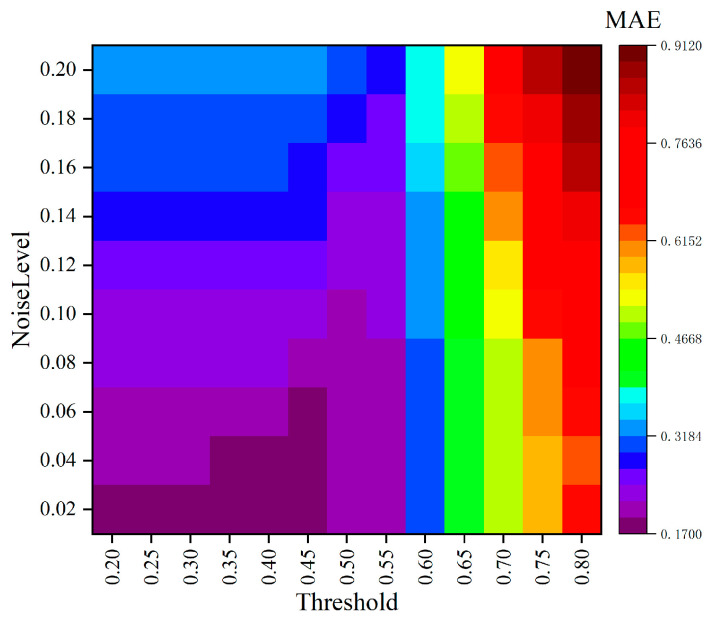
Distribution of the MAE of the reconstructed results for different noise levels and threshold values.

**Figure A5:**
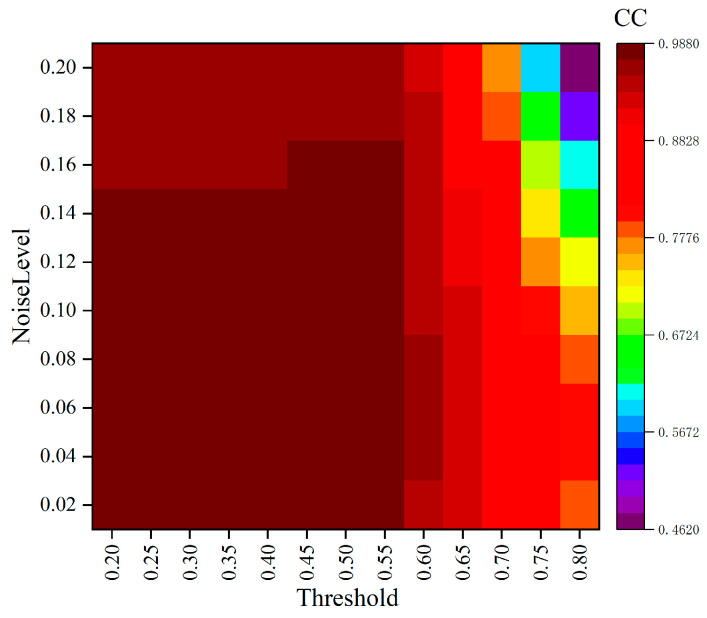
Distribution of the CC of the reconstructed results for different noise levels and threshold values.

**Figure A6:**
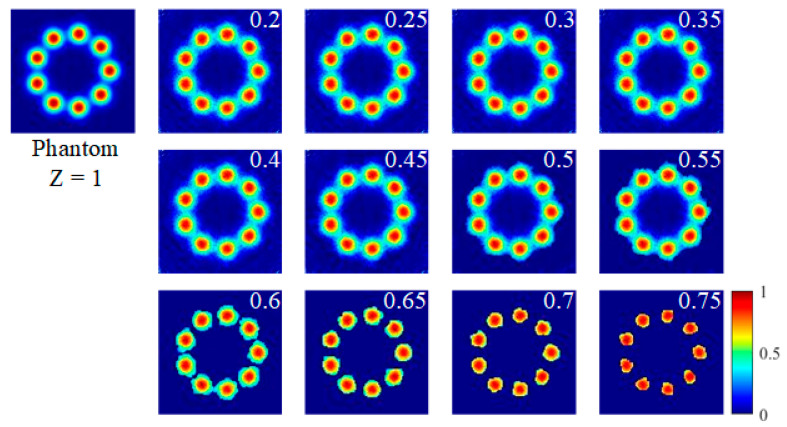
Reconstructed slices at different threshold values for a noise level of 0.04.

**Figure A7:**
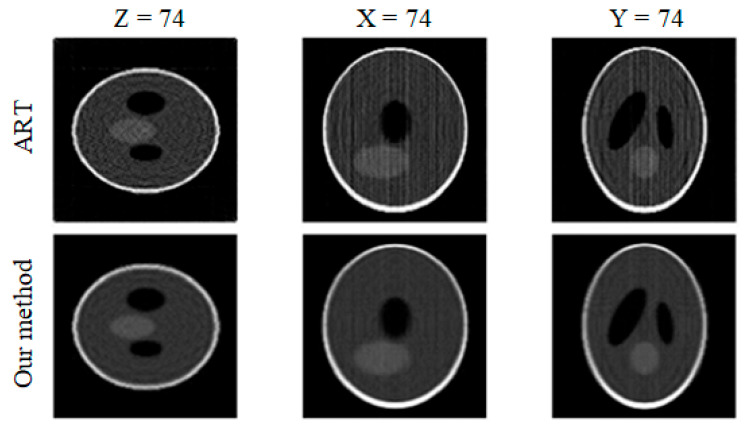
Comparison of reconstruction results slices between ART and the threshold-constrained LFBP-SART.

**Figure A8:**
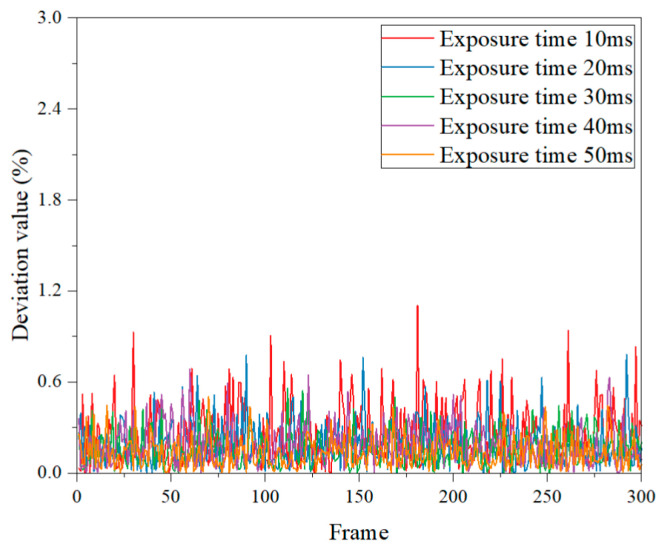
Standard grayscale response value of camera imaging over time at different exposure times.

**Figure A9:**
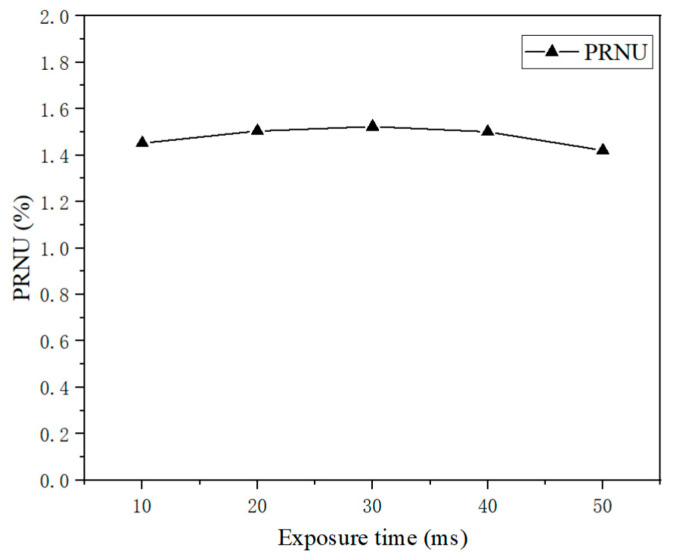
PRNU under different exposure times.

## Data Availability

The original contributions presented in this study are included in the article. Further inquiries can be directed to the corresponding author.

## Appendix A

### Appendix A.1

A numerical simulation of the modified LFBP-SART reconstruction was performed to evaluate the influence of the number of views. Three-dimensional annularly distributed Gaussian functions are employed to simulate the radiation distribution of the Bunsen burner’s small flame, as shown in Figure A1. The phantom domain was discretized into 100 × 100 × 40 voxels, and 36 2D projections were generated by Radon transform, with uniform random noise added to the projection data to model the noise introduced by camera. Reconstruction was then performed using 9, 16, and 36 uniformly distributed views, and the reconstructed fields were compared with the ground-truth phantom to quantify the reconstruction error.

The reconstruction results with the modified LFBP-SART algorithm are presented in Figure A2 and Figure A3. The correlation coefficient (CC) and mean absolute error (MAE) were computed for evaluation, and the values are shown in Table A1.

**Table A1:** MAE and CC of reconstruction result of different view numbers.

View Numbers	9	18	36
**MAE**	0.1548	0.0925	0.0629
**CC**	0.9890	0.9959	0.9982

The simulation and evaluation results demonstrate that when the number of projections exceeds 18, the reconstruction results obtained using the modified LFBP-SART algorithm accurately restore the original distribution, with a CC greater than 0.99 and a MAE less than 0.1. In this work, we use 36 views to reconstruct the OH* distribution of the Bunsen burner to ensure reconstruction accuracy.

### Appendix A.2

To determine an appropriate threshold value for the modified LFBP–SART algorithm during reconstruction, we further evaluated the reconstruction quality under different noise levels using the 3D annularly distributed Gaussian phantom. In this study, 36 projection views were used for reconstruction, and different levels of additive noise were superimposed on the simulated projections. The noise level is defined as the ratio of the mean absolute noise amplitude to the mean value of the projection data. Figure A4 and Figure A5 shows the corresponding heat maps of MAE and CC as functions of the threshold and noise level. When the noise level in the multi-view projection data is relatively low, a smaller threshold allows weaker image features near the edges to be preserved to the greatest extent; however, an overly small threshold leads to an increase in reconstruction artifacts. As an example, at a noise level of 0.04, Figure A6 shows the reconstructed slice at Z = 1 for different threshold values. For smaller thresholds, the reconstructed annular Gaussian distribution agrees well with the ground-truth phantom, but a large number of noise-induced artifacts appear in the regions surrounding the ring, especially near the image boundaries. As the threshold increases, these artifacts are significantly reduced. At the same time, if the threshold is too large, the reconstruction preserves high-intensity voxel structures but progressively suppresses and eventually removes low-intensity voxel details. Therefore, considering the above trade-offs between artifact suppression and detail preservation, we recommend choosing a threshold in the range 0.45–0.55 for practical reconstructions.

### Appendix A.3

To compare the reconstruction performance of the threshold-constrained LFBP-SART and ART algorithms, we conducted numerical simulations using a head phantom with rich internal 3D structures. The head phantom has a size of 128 × 128 × 128 voxels, and 2D projections were generated in 36 directions using the Radon transform. Uniform additive noise was superimposed on the projections. The MAE of the ART and modified LFBP-SART reconstructions are 0.3826 and 0.3064, respectively. Slices of the reconstructed volume are shown in Figure A7. In the ART reconstructed slices, pronounced streak artifacts were observed, whereas in the threshold-constraint LFBP-SART results these artifacts were significantly suppressed.

## Appendix B

### Appendix B.1

The Radiometric calibration is conducted in National Institute of Metrology, China, which is responsible for ensuring nationwide consistency and international equivalence of quantity values, maintaining the highest measurement capabilities in China. The relative uncertainty and accuracy class of the calibration instruments is shown in Table A2. The relative uncertainty in the spectral range of 250–400 nm is less than 3%.

**Table A2:** The relative uncertainty and accuracy class of the calibration instruments.

Instruments	Spectral Range (nm)	Relative Uncertainty (*U*_rel_) /Accuracy Class (*k*)	Certificate Number
**White board**	250–2500	250–380 nm, 780–2500 nm: *U*_rel_ = 1.0% (*k* = 2); 380–780 nm: *U*_rel_ = 0.44% (*k* = 2)	GXcl2023–08027
**Spectral irradiance standard lamp**	250–2500	250–400 nm: *U*_rel_ = (3.0–1.3)% (*k* = 2); 400–800 nm: *U*_rel_ = (1.3–1.2)% (*k* = 2); 800–2500 nm: *U*_rel_ = (1.2–3.5)% (*k* = 2)	GXfs2024–00332

### Appendix B.2

The Prime 95B CMOS camera is a UV back-illuminated enhanced CMOS camera with a response band ranging from 200 to 1000 nm. The temporal stability and spatial uniformity of the camera were assessed as follows.

To analyze the stability of the camera over time, 300 frames of continuous images were acquired for the standard light source under different integration times (10 ms, 20 ms, 30 ms, 40 ms, 50 ms). We define standard grayscale response as the mean grayscale value of a 10 × 10 area in the center of the FOV. The deviation of the standard grayscale response at different integration times was calculated as follows:

(A1) $$D_{t_{i}}=\left|\frac{G_{t_{i}}-G_{t_{avr}}}{G_{t_{avr}}}\right|$$

where $D_{t_{i}}$ is the deviation value at time *t_i_*, $G_{t_{i}}$ is the standard grayscale response value at time *t_i_*, and $G_{t_{\mathrm{avr}}}\;$ is the average of the standard grayscale response values of 300 frames. As shown in Figure A8, the deviation values under different integration times (10 ms, 20 ms, 30 ms, 40 ms, 50 ms) are less than 1.2%.

To quantitatively describe the spatial uniformity of camera imaging, Photo Response Non-Uniformity (PRNU) was calculated as the ratio of the standard deviation of each pixel’s output signal to the average signal, as follows:

(A2) $$PRNU=\frac{1}{\bar{G}}\sqrt{\frac{1}{xy}\sum_{x=1}^{x}\sum_{y=1}^{y}{\left(G(x,y)-\bar{G}\right)}^{2}}$$

Figure A9 shows the PRNU under different exposure times, which is primarily distributed between 1.4% and 1.6%. The results meet the EMVA 1288 standard, which states that the spatial non-uniformity of a qualified sensor should be less than 3%. Based on these results, the UV camera has good temporal stability and spatial uniformity, which ensures accuracy for the quantitative measurement of free radical concentration.
